# Climate-resilient health systems in the face of extreme weather events in Uganda: a qualitative study in three high-risk districts

**DOI:** 10.1186/s12913-026-15326-z

**Published:** 2026-08-10

**Authors:** Rawlance Ndejjo, Daniel Hellden, Susan Karuhanga, Irene Wanyana, Charles Batte, Nina Viberg, Angela Y. Chang, Robert Marten, Tobias Alfven, Rhoda K. Wanyenze

**Affiliations:** 1 Department of Disease Control and Environmental Health, School of Public Health, Makerere University, Kampala, Uganda; 2 Institutes for Transdisciplinary Scholarships, University of Calgary, Calgary, Canada; 3 Department of Global Public Health, Karolinska Institutet, Stockholm, Sweden; 4 Department of Epidemiology and Biostatistics, School of Public Health, Makerere University, Kampala, Uganda; 5 Department of Medicine, Lung Institute, School of Medicine, College of Health Sciences, Makerere University, Kampala, Uganda; 6 Danish Centre for Health Economics, University of Southern Denmark, Odense, Denmark; 7 Danish Institute for Advanced Study, University of Southern Denmark, Odense, Denmark; 8 Alliance for Health Policy and Systems Research, World Health Organization, Avenue Appia 20, Geneva, 1211 Switzerland; 9 Sachs’ Children and Youth Hospital, Stockholm, Sweden; 10 Department of Preventive Medicine, College of Medicine, Korea University, Seoul, South Korea

**Keywords:** Adaptation, Droughts, Extreme weather events, Floods, Landslides, Health systems, Uganda

## Abstract

**Background:**

Uganda’s climate is changing with an increase in the frequency and intensity of extreme weather events including droughts, floods, heatwaves, and landslides, with potential impact on health systems. This study documented how these events have impacted health systems and their resilience in three highly vulnerable districts of Bududa, Kasese and Moroto in Uganda, to inform strategies for building climate-resilient health systems in sub-Saharan Africa and similar settings.

**Methods:**

This study employed a case study approach using qualitative interviews among key informants who were purposively selected. A total of 55 interviews were conducted among stakeholders at the national and district levels as well as non-governmental organizations, including health and non-health officers, health workers, program managers, directors or unit heads. Interviews were audio-recorded and transcribed verbatim. Study transcripts were deductively coded considering the impacts on the health system and adaptation measures in line with the World Health Organization’s Operational framework for climate-resilient and low-carbon health systems. Study quotations were added to supplement the study findings.

**Results:**

The study established that landslides, floods, and droughts impacted health systems in the three districts through difficulty coordinating multiple players during response, causing injuries and deaths among health workers, damaging infrastructure, drugs, and information systems. These impacts affected health services delivery. To adapt, the health system employed several strategies including instituting policies, early warning systems, capacity building for health workers, and increasing stock of medicines and supplies at district stores. The other mechanisms were improving water and sanitation at facilities, relocating some facilities, engaging the community on potential diseases and risks, and mobilizing funding to support response. Overall, factors such as a clear policy framework including mechanisms for stakeholder coordination, political will, availability of resources and health workers, high community engagement, and robust surveillance and strong early warning systems were key enablers for the health system adaptation.

**Conclusions:**

Extreme weather events impacted all facets of the health system, and districts undertook various mechanisms to deal with them. These lessons should be integrated into strengthening the capacity of health systems to anticipate, respond to, cope with, and recover from extreme weather events.

**Supplementary Information:**

The online version contains supplementary material available at https://doi.org/10.1186/s12913-026-15326-z.

## Introduction

As the world’s climate changes, countries in sub-Saharan Africa are increasingly vulnerable to its effects [1, 2]. Uganda’s climate is changing with the average temperature in semi-arid areas rising and the frequency of hot days increasing [3, 4]. These changes have contributed to significant extreme weather events, including increased frequency and intensity of droughts, floods, heatwaves, and landslides [3, 4]. Floods, landslides, and droughts are among the most predominant extreme weather events in Africa [5, 6]. The eastern part of Uganda has experienced extreme rainfall conditions, while the western, northern, and north-eastern regions have had more frequent, severe, and longer-lasting droughts since 2000 than previously reported [4]. The impacts of these extreme weather events have been enormous on the population, contributing to disease spread and mortality. For example, droughts, floods and landslides have aggravated the occurrence of water-borne diseases such as dysentery, cholera, hepatitis E; vector-borne diseases especially malaria; respiratory diseases; and malnutrition-related illnesses [7–10]. Furthermore, these events have led to displacement of populations, food insecurity, environmental degradation, reduced agricultural production and productivity, and damage to infrastructure [9, 11, 12]. These impacts have also been more severe among vulnerable and marginalized populations including children, women, the poor, older persons, and people with disabilities [13, 14].

Across sub-Saharan Africa, health systems are fragile, weak, and inadequately funded [15, 16] with less potential to contain shocks and stressors due to emergencies and climate change as demonstrated during the COVID-19 pandemic. As extreme weather events increase in occurrence and intensity due to the impacts of climate change, it is imperative to build climate-resilient health systems. These health systems can ‘anticipate, respond to, cope with, recover from and adapt to climatic shocks and stresses, to bring sustained improvements in population health’ [17]. As an important first step, health policy and systems research should systematically document the lessons and experiences of how health systems in areas that routinely experience climate shocks have adapted and mitigated these events [18]. A few local studies have highlighted how extreme weather events have impacted health outcomes [9, 19, 20], however, there hasn’t been an in-depth examination of the impact on the entire health system. Drawing from multiple disciplines and using a systems lens, health policy and systems research is well positioned to not only document the interlinkages between climate change, health, and health systems but also propose appropriate response measures [18]. This study documented how extreme weather events have impacted health systems and their resilience in Bududa, Kasese and Moroto districts in Uganda to inform strategies for building climate-resilient health systems in sub-Saharan Africa and similar settings.

## Methods

### Study areas

**Fig. 1:**
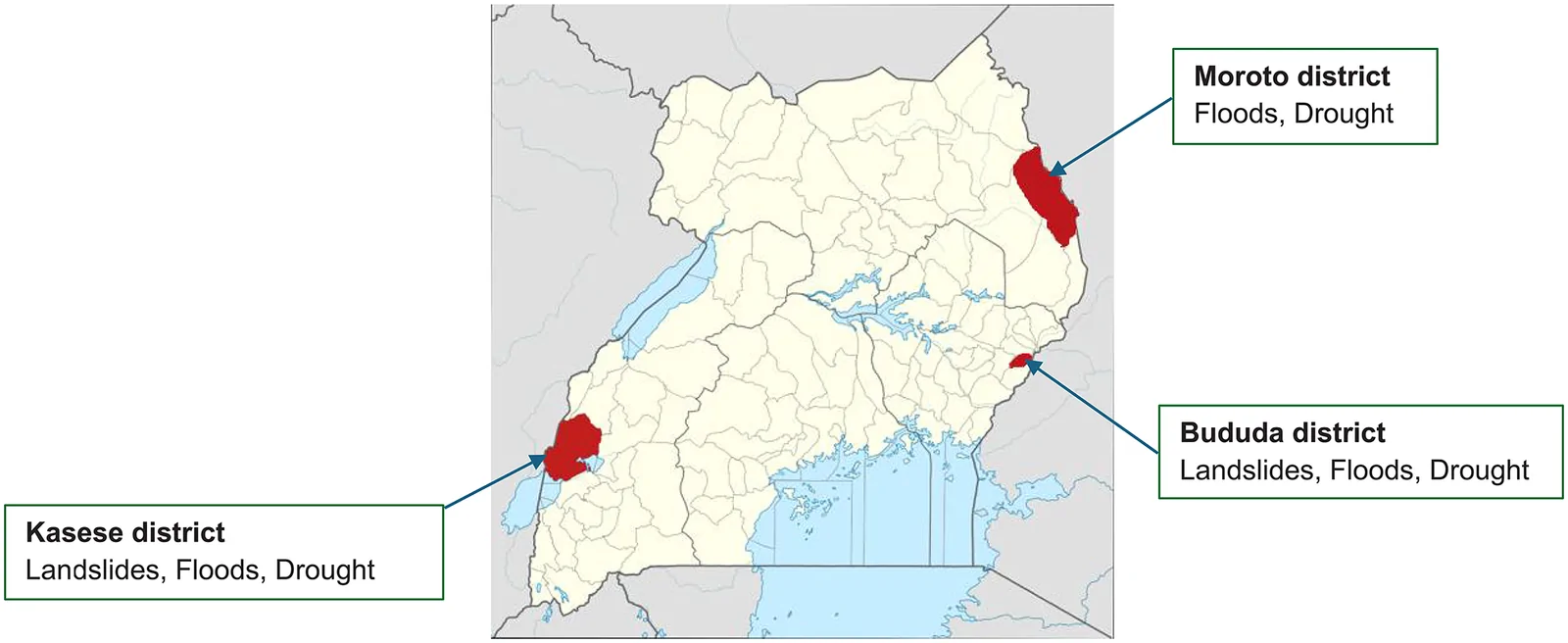
Map of Uganda showing the study districts and the key extreme weather events

This study was conducted in Bududa, Kasese, and Moroto districts in Uganda. These districts are among those that frequently experience extreme weather events, especially floods, landslides, and droughts. In Bududa district in Eastern Uganda on the slopes of mountain Elgon, heavy rains usually lead to landslides causing deaths, property loss, and massive population displacement. In the aftermath, affected communities register higher mortality and morbidity rates, injuries, unsafe water, sanitation and hygiene situations, and poorer livelihoods [21–23]. In the south-western district of Kasese, rainy seasons usually come along with flooding causing deaths, damage to infrastructure, and population displacement. In 2020, over 120,000 people were displaced by floods in Kasese district. Flooding in the district has increased the risk of malaria [9], typhoid fever [19], and cholera outbreaks [20] overwhelming the health system. The mid-northeastern district of Moroto in the Karamoja sub-region experiences drought and prolonged dry spells, strong winds, floods, and bushfires attributed to extreme weather events. The district suffers chronic food insecurity and malnutrition [24] due to the combined impacts of high poverty levels, low human development index, and extreme climate and weather conditions. Bududa, Kasese and Moroto are largely rural and remote and access to health care is plagued with multiple challenges including drug and equipment shortages, inadequate qualified human resources, and poor quality of health services. The districts are also hilly and mountainous with a poor road network and distances to facilities are long. Figure 1 shows the location of the study districts and the key extreme weather events they face.

### Study design, population, and sampling

This study adopted a case study design and employed qualitative interviews among key informants (KI) who were purposively selected due to their expert knowledge and awareness of climate change adaptations at different levels, national, district and sub-district. Stakeholders were selected from multiple sectors to document a wide array of measures beyond a single sector. These sectors included health, environment, agriculture, education, water, sanitation and hygiene, and development. The stakeholders were from government, private sector, civil society, and international organizations at national and district levels and included national and district health and non-health officers, health workers, program managers, and organization directors or unit heads. A total of 55 interviews (45 targeting district stakeholders and ten among national-level actors) were conducted informed by the principle of saturation, which was examined during daily debrief meetings.

### Data collection

Data collection was informed by a pretested key informant interview guide (Additional file 01) developed in line with the World Health Organization (WHO)’s Health Systems Building Blocks and the Operational framework for climate resilient and low carbon health systems [25]. The interview guide had three main themes including the impact of extreme weather events on the health system, the health system adaptation mechanisms, and the barriers and facilitators of building health systems adaptation and resilience. Interviews were conducted in English at the offices of the selected key informants or other suitable places by trained and experienced research assistants. The research assistants were six in total, four of whom were females and all had bachelor’s level training in health and development fields with experience conducting qualitative research. Interviews and discussions were audio-recorded. The data collection process was overseen by two researchers (RN-male) and SK (female) both with training in health sciences and vast experience in conducting qualitative studies.

### Data management and analysis

The recorded interviews were transcribed verbatim in English. The transcripts were checked and verified by another team member while listening to some of the original audio recordings. We conducted a Microsoft-Excel based thematic matrix analysis with deductive themes informed by the study frameworks. Specifically, two researchers read the study transcripts and developed a draft codebook that was discussed and harmonized with the study team. The researchers then coded the findings relating to the impacts of extreme weather events on the health system in line with constructs from the WHO Health Systems Building Blocks and mitigation and adaptation measures undertaken according to the WHO Operational framework for climate resilient and low carbon health systems [25]. During coding, newer codes were discussed among the coders and routinely added to the codebook. Where the coders did not reach agreement, a third researcher was engaged. Following coding, similar codes were merged into sub-themes. Quotations from study participants were added to supplement the study findings. Reporting for this study was informed by the Consolidated criteria for reporting qualitative research (COREQ) guidelines (Additional file 02).

## Results

### Impact of extreme weather events on the health system

Landslides, floods and drought, the major extreme weather events in the three districts, impacted the health system across its six building blocks as described below and summarized in Table 1.

#### Leadership and governance

As different sectors and levels of government come together to address the extreme weather events, coordination of multiple players becomes cumbersome and ineffective, slowing down planning, decision-making, and response interventions. There was a noted diversion of focus from planned health activities to the emergencies caused by extreme weather events. The process of dealing with extreme weather events also created conflict among stakeholders as they planned, engaged and strategized to address the health needs of the affected communities. Political interference in the management of health services in the affected districts was also reported with security and political interests sometimes overriding those of health affecting autonomy. Politics was also extended to the aftermath of the weather event such as with politicians influencing the location of newer health facilities to claim credit with some disputes ending in court as registered in Bududa district. In Moroto district, it was noted that immigration increased, including from the neighboring Turkana region in Kenya, heightening insecurity, as local and migrant communities moved in search of water and pasture for their animals in aftermath of droughts. Increased immigration aggravated service delivery pressures and led to increased emigration of locals to neighboring districts to avoid competition for resources and conflict.

*We are unable to plan properly and meet targets because we don’t know what is going to happen. Even when you predict based on your earlier knowledge*,* it is surpassed by another extreme event. So*,* planning becomes very difficult as well as delivery since you operate in crisis mode with elevated costs. Sometimes there is pressure to keep moving services to where the population is migrating to*,* such as*,* during a drought. So*,* they say*,* ‘our people have moved*,* take a borehole there or go and immunize them’. But we know they will move away again. So*,* you can’t plan like that for a very mobile community which is suffering from repeated shocks.* (Administrator, non-health sector, Moroto district)

#### Health workforce

Extreme weather events led to injuries and/or death among health workers. As health facilities become hard to access, the availability of health workers is impacted. Other health workers also leave their posts in search of opportunities elsewhere thus affected districts usually record high attrition rates. These factors contribute to increased absenteeism, workload and disrupted shift schedules among health workers at the facility as noted by one health worker, “*Health workers attendance at the facility will tend to drop because they will have the excuses of extreme weather conditions*,* and most of them will report being sickly. The workload will then be left to the few who will be at the facility*.” The aftermath of the disaster often comes with increased morbidity in the population, especially from infectious diseases which overwhelm health facilities and health workers. The health and well-being of health workers is also affected by the enhanced fear, distress, and stress of working in areas deemed unsafe.

*During the last flooding event*,* health workers struggled to get to the facilities and even while there*,* they were fearful that whenever it threatened to rain*,* they would close and go away*,* and when community members went to the facility*,* they would find it closed*. (Health worker, Kasese district)

#### Health information systems

Health facilities also experienced interruptions to information systems due to difficulty in accessing health facilities to keep up with the documentation, disruptions in electricity supply, destruction or loss of facility data, and damage to equipment. The increased workload at the facility or in the nearby communities, and the emergency nature of the response limited prioritization of data entry. The affected communities also lose or leave their health records behind impacting their access to health care, retrieval of records, and data completeness at the facility. Health workers also struggle to use web and mobile-based data collection tools in locations with poor internet and mobile phone network. These factors contribute to data inaccuracies, incompleteness, and untimeliness thus leading to poor data quality.

*Our performance is affected because that [health information] system is based on certain indicators which are tracked routinely. So*,* when you produce your report*,* you’ll find some serious outliers. You’re targeting 200 people for vaccination and suddenly you go and vaccinate 350 and the coverage is over 100% and other places register only 30%. Then people will say the health department is not planning well. And then in some places where you have web-based or online tools*,* you may not find a network signal to register the information you are collecting.* (Administrator, health sector, Moroto district)

#### Essential medical products and technologies

Landslides and floods were reported to damage infrastructure and lead to the loss of medical products and technologies impacting stock available at facilities. A health worker in Kasese noted that “*Currently*,* we have no X-ray machine as it got damaged during the floods. So*,* we just refer patients in need of this service*.” The high temperatures that come with drought were also noted to affect the storage of medicines and supplies including vaccines and complicate their transportation for outreaches and their ultimate potency. The increased demand for health care in the aftermath of weather events also means a stretching of the supplies to deal with the increased disease burden and vulnerability of the community compounding stockouts of supplies. To exacerbate the situation, the damage to infrastructure including roads hampers the delivery of supplies and the duration of stockouts increases.

*It is always a challenge delivering essential medicines and supplies to the community because of their inaccessibility following heavy rains and floods. The roads are cut off*,* are slippery*,* and the vehicles are unable to pass. So*,* you will find that*,* at times we have stockouts at the facility*,* yet the medicines are within the district*. (Health worker, Bududa district)

#### Service delivery

Landslides and floods led to the destruction of infrastructure including health facilities, roads, and bridges thus increasing difficulty in access to health services with vulnerable groups such as pregnant women, the elderly, and people living with disabilities heavily impacted. Droughts were mentioned to drive away communities to far areas that have favorable weather, but much further from health facilities, increasing difficulties in access to health care. These impacts together with the effects on health workers contribute to reduced care seeking yet affected communities register an increased demand for health services. In Moroto for example, malnutrition was noted to increase during drought periods together with the demand for supplements. In Kasese, flooding damaged health facilities and health services were relocated to other facilities, further from the catchment community increasing healthcare access difficulties. Moreover, referral systems are negatively impacted due to the damaged infrastructure. Other ongoing facility-based and outreach service delivery activities including immunization programs for children are also affected. The quality of care is also negatively impacted by the increased healthcare demand amidst limited supplies and overwhelmed health workers. In some instances, the aftermath of the event and the damage to facilities led to them being downgraded to lower levels, impacting the range of services offered. One hospital in Kasese was downgraded to a lower-level health facility offering lesser services.

*Floods have affected the hospital which serves around six sub counties that have only two health centers. So*,* patients normally fail to get where to access healthcare when the river floods. Previously we recorded two major flooding events*,* and the hospital was relocated to the town center*,* more than 11 kms away. And now if you add the distance from where these patients come from in those mountainous areas*,* it becomes very long without any reliable transport mechanisms. The transportation of patients from the mountains to this valley is by stretchers*,* and carrying those stretchers from here to the town is not easy. Thus*,* many people have been dying in the process of going to the hospital because of the far distance due to the washing away of the hospital in its previous location*. (Health worker, Kasese district)

#### Financing

Extreme weather events come with heightened pressure for financial resources which usually deplete the district health resources and yet the process of accessing additional resources from the government was lengthy and not guaranteed. The destruction of health infrastructure, renovations and/or construction of temporary health facilities greatly impacts the limited available resources. Transportation of medical products also becomes more costly as longer routes are used to avoid the affected roads and bridges. The district actors are thus expected to mobilize finances from various sources to support the health system and fill in emerging gaps diverting their attention from their usual roles.

*It [extreme weather events] put a burden on us to look for resources for rehabilitation of infrastructure*,* sensitization of communities*,* and sometimes even replacement of the affected facilities. We also need resources for equipping staff and supporting emergency delivery of medicines and supplies*. (Community-based services, Bududa district)

**Table 1:** Impact of extreme weather events on the health system

Health system building blocks	Impacts of extreme weather events
Leadership and governance	• Difficulty in coordinating multiple players. • Conflicts with security and political interests. • Increased insecurity and immigration in the community. • Diversion of attention and resources from other health system issues to dealing with the weather event.
Health workforce	• Injuries/death of health workers. • Unavailability of health workers. • High attrition of health workers. • Increased absenteeism among health workers. • Increased workload for health workers. • Disrupted health worker shift schedules. • Negative impact on health and well-being of health workers.
Health information systems	• Interruptions to information systems reporting. • Reduced data quality. • Over or underestimation of statistics. • Compromised infection prevention and control mechanisms.
Essential medical products and technologies	• Loss of medical products and technologies. • Elevated demand for supplies. • Increased difficulty delivering supplies. • Loss of potency of medicines and vaccines.
Service delivery	• Destruction of health facility infrastructure. • Disruption of transportation infrastructure increases difficulty accessing health services. • Reduced availability of health services. • Increased demand for health services. • Breakdown of referral systems. • Disruption of health programs. • Reduced quality of care. • Downgrading of health facilities. • Crowding from acting as shelter for displaced persons.
Financing	• Depletion of district health budgets. • Diversion of resources from other facility activities. • Pressure to mobilize resources to support response.

### Health systems adaptation to extreme weather events in the three districts

Several initiatives and activities were implemented in the three districts to increase the health system’s capacity to anticipate, prepare for, adapt, and recover from the negative impacts of extreme weather events. These initiatives are described below and summarized in Table 2 following the ten components of the WHO Operational framework for climate resilient and low carbon health systems.

**Table 2:** Health system adaptation mechanisms in Bududa, Kasese and Moroto districts in Uganda

Health system building blocks	Climate resilient and low carbon systems component	Adaptation mechanisms (anticipate, prepare, adapt, and recover)
Leadership and governance	Climate transformative leadership and governance	· A comprehensive legal and policy framework that supports adaptation with a clear mandate of the health sector. · Disaster management policy and disaster risk contingency plans to support response. · Establishment of district disaster committees for joint planning with heads of departments and their decentralization to selected sub-units. · Mapping partners and their functions in preparedness and response. · Participation of health administrators in disaster committees. · Advocating for increased funding for climate change and health programs.
Health workforce	Climate-smart health workforce	· Capacity building and training of health workforce on the intersection between climate change and health. · Increasing awareness of climate change and health risks among health workers. · Incentivizing health workers who work in affected areas such as accommodation, transport, and/or additional allowances. · Increasing staffing levels at health facilities. · Empowerment of lower-level health units to strengthen their capacity and scope of services. · Posting disaster-familiar staff to prone areas. · Recall of health workers off duty to support response and/or reassignment of those from other facilities. · Psychosocial support for health workers. · Close supervision to ensure attendance of health workers at hard-to-reach facilities.
Health information systems	Assessments of climate and health risks and greenhouse gas emissions	· Joint assessments after extreme events to determine response and for future planning. · Assessing vulnerabilities of health facilities and communities to inform interventions.
	Integrated risks monitoring, early warning, and greenhouse gas emissions tracking	· Monitoring risks among vulnerable communities and health facilities to inform action including relocations. · Developing and reviewing early warning systems. · Surveillance of high-risk areas for disease outbreaks. · Provision of information to communities and leaders as part of the early warning system. · Engaging communities to report early signs of extreme weather events. · Education of communities on potential diseases and risks.
	Health and climate research	· Encouraging research on the intersection of climate change and health. · Close engagement with researchers to inform the research. · Creating mechanisms to support the uptake of climate resilience innovations by health systems.
Medical products, vaccines and technologies	Climate resilient and low carbon infrastructures, technologies, and supply chain	· Equipping district stores with drugs and increasing availability of emergency drugs. · Implementation of climate-resilient infrastructure and designs including maintenance of road infrastructure to facilities. · Bolstering health facility infrastructure including construction of drainages. · Locating new facilities in safer areas. · Use of alternative means to support last-mile delivery including heavy-duty vehicles, motorcycles or manual carrying of drugs. · Increase supplies including through working with neighboring districts and implementation partners. · Revising and adjusting stock based on expected health risks of extreme weather events. · Increasing access to utilities including electricity and water at facilities and creating reserve storage or alternatives. · Stocking emergency supplies such as water purification tablets and oral rehydration salts.
Service delivery	Management of Environmental determinants of health	· Provision of energy and safe water and sanitation to facilities.
	Climate-informed health programs	· Increasing outreaches on preventable infectious diseases to reduce their risk of spread during disasters. · Community sensitizations on climate and health risks. · Community engagement on climate and health risks. · Psychosocial support to communities.
	Climate-related emergency preparedness and management	· Upgrading facilities from health centers to hospitals · Designation of evacuation centers and facilities to handle specific conditions. · Designating or repurposing spacious wards to receive clients. · Standard operating procedures to respond to environmental risks. · Training lay staff at facilities on handling emergencies and evacuating affected people. · Attaching essential equipment including ambulances to facilities. · Increasing geographical accessibility to health facilities and services. · Linkage with traditional birth attendants in the communities to strengthen referral. · Improvisation of alternative transport means for health workers to get to facilities including motorcycles.
Financing	Sustainable climate and health financing	· Anticipatory budgeting integrating climate change planning in health budgets. · Diversion of resources and equipment to support the health system. · Increasing funding for climate change and health programs. · Mobilization of resources and funds from stakeholders including non-governmental organizations and the private sector.

#### Climate transformative leadership and governance

The health system implemented several measures at the leadership and governance level to mitigate the effects of extreme weather events. The highlighted measures were: the legal and policy frameworks with a clear mandate of the health sector, the integration of health in climate change planning, the role of district disaster management committees, and disaster risk contingency planning. In the districts, the disaster committees were extended to lower sub-units in vulnerable areas including sub-county and village levels to support preparedness. The district stakeholders also advocated for increased funding to support climate change and health programs, and in Moroto district, some stakeholders mentioned mapping organizations and stakeholders who support implementation and their functions in preparedness and response in advance to ease coordination and collaboration.

*We try to mobilize*,* and coordinate partners as one of the strategies to respond quickly because partners have resources which are more flexible. So*,* we map partners*,* we map those who can respond to emergencies and those for normal programming. So*,* in the event of extreme weather events*,* we know where to start from.* (Administrator, non-health sector, Moroto district)

#### Climate-smart health workforce

Initiatives included capacity building and training the health workforce on the intersection between climate change and health, providing additional incentives to health workers in affected areas. The training focuses on the health impacts of extreme weather events including vector-borne diseases such as malaria, water-borne diseases such as cholera and food insecurity and malnutrition as well as adaptive response at facility and community levels. The incentives included the provision of accommodation for health workers at the facility to reduce travel and increase their availability at facilities or availing transport and/or additional allowances. The other measures were increasing staffing at facilities to increase availability at facilities which was reported in all districts and posting disaster-familiar staff to the prone areas which was noted in Bududa district. During response, health workers off duty are also recalled and those from other areas are reassigned to support affected facilities. Health leaders mentioned providing continuing psychosocial support to health workers. This took the form of peer support, counselling, self-care strategies and specialized mental health services to workers based on need. There is also close supervision to ensure the attendance of health workers at hard-to-reach facilities especially to support recovery.

*During the planning and budgeting process*,* we are very mindful of the areas which are in hard-to-reach areas*,* so we tend to plan for staff accommodation to reduce staff commuting every time in the morning or evening. Sometimes when it rains a lot*,* health workers may not report for duty because maybe the road is cut off but if someone is already there at the facility*,* work can continue with fewer interruptions. *(Community-based services, Bududa district)

#### Assessments of climate and health risks and greenhouse gas emissions

Actors and districts reported conducting joint assessments in the aftermath of extreme events to determine response, plan for future response and a vulnerability assessment of facilities and communities to support tailoring interventions to them. Many facilities in the districts reported undergoing a vulnerability assessment, most times by external actors. However, they reported limited action based on the findings.

#### Integrated risks monitoring, early warning, and greenhouse gas emissions tracking

The districts strengthened their early warning systems and mechanisms to enhance predictions of extreme weather events and provide tailored information to facilities and communities. For example, districts reported engaging in participatory scenario planning based on their past experiences and being equipped with automated weather stations, flood detection equipment, and rain gauges among others to inform forecasting. A health worker in Kasese district noted that “*Here the river floods between the month of May and July*,* so we now got used and begin our preparations in May*.” The districts also conduct surveillance of high-risk areas for disease outbreaks and provide relevant information to communities to support preparedness and minimize health system impact. Information flow with the community is also two-way providing key information to district leaders to support forecasting and predictions of extreme weather events including based on indigenous knowledge. To support response and recovery, the district and facilities together with other stakeholders also conducted community engagement on potential diseases and risks to promote health.

*In the Karamoja region*,* we have provided flood detection equipment which monitors the water levels. Once we estimate the amount of anticipated rain is above the flood threshold*,* then we can provide an early warning signal for stakeholders to prepare for flooding. *(Programming, Nongovernmental Organization, Moroto district)

#### Health and climate research

Stakeholders reported efforts to encourage research at the intersection of climate change and health with the districts and facilities engaging with researchers to inform their research and ensure it is tailored to their needs. Mechanisms to support the uptake of climate resilience innovations by health systems were also reported. However, they noted a gap in translating knowledge on climate and health research into policies and actions towards building climate change-resilient health systems.

#### Climate-resilient and low-carbon infrastructures, technologies, and supply chain

The adaptation measures included implementation of climate-resilient infrastructure and designs for new facilities and ensuring that they are in safer areas. For existing facilities, efforts are geared towards renovating their infrastructure and improving drainage. The authorities also work towards increasing access to utilities including electricity and water at facilities and creating a reserve storage or alternative that can support during crisis times. Some districts mostly with support from implementing partners provided alternative energy sources such as solar energy to support facilities. To increase the availability of supplies, there have been efforts to equip district storage facilities with drugs especially emergency drugs to ease their distribution to facilities in cases of need. To support the distribution, district efforts have also focused on the maintenance of road infrastructure to facilities to improve geographical access.

*This district is susceptible to weather vagaries*,* so we maintain stock of some drugs especially Oral rehydration salts*,* zinc and water treatment tablets so that in case of floods or population displacement*,* we can ably give water treatment tablets*,* and the population gets clean water.* (Health services, Kasese district)

During response to extreme weather events, health facilities reported the use of heavy-duty vehicles to ensure last-mile delivery or alternative transportation means including motorcycles or manual carrying of drugs. They also worked together with neighboring facilities, districts and non-governmental organizations to increase supplies to affected facilities. Following events, there is a review of supply processes to revise and adjust stock based on realities posed by the health risks of extreme weather events.

#### Management of environmental determinants of health

To support the management of environmental determinants of health, most efforts were geared towards the provision of safe water and improvement of sanitation infrastructure at health facilities. This was mostly due to water scarcity that is registered during droughts or contamination during floods reducing accessibility to safe water.

*Our water*,* sanitation*,* and hygiene project has motorized boreholes in health centers because during the dry season*,* the water table in Karamoja goes very low and water becomes scarce. With this intervention*,* if a mother comes to the facility*,* they can have safe water for washing or drinking. In other facilities*,* we have provided a rainwater harvesting tank in health centers*. (Programs, Non-governmental Organization, Moroto district)

#### Climate-informed health programs

The facilities mostly worked with stakeholders, including non-governmental organizations, to increase outreaches targeted at preventable infectious diseases to reduce their risk of spread during disasters. These outreaches also involved education programs to empower the community about climate risks and disease spread, also supported by community resources such as local leaders and community health workers. In some areas, community dialogue events were usually organized to interact and share information such as on gardening, food preservation and water harvesting in anticipation of drought. Psychosocial support, mostly in the form of counselling and mental health services, is offered to communities that have experienced extreme weather events. At the health facility, clients are also sensitized about the health risks of concern and offered psychosocial support.

*We do a lot of community engagement. We talk to them [the community] and even go as far as doing demonstrations such as on kitchen gardening and tell them*,* ‘This is how you can support if the rain is not there’. We also teach mothers who come here with their children for outpatient therapeutic care. We teach them how to establish a small garden*,* water it*,* and plant vegetables which they can harvest and eat or sell for money*. (Health worker, Moroto district)

#### Climate-related emergency preparedness and management

Measures to support preparedness and management included upgrade of some facilities from health centers to hospitals to widen their range of services, designating or repurposing spacious facility wards to receive clients, and attaching essential equipment including ambulances to facilities. Facilities also created standard operating procedures to respond to environmental risks and trained lay staff, including community health workers and drivers, on handling emergencies and evacuating affected people. Other efforts focused on increasing geographical accessibility to health facilities and services. During response, some facilities utilized their linkage with traditional birth attendants in the communities to encourage them to refer mothers to health facilities.

#### Sustainable climate and health financing

In terms of financing, the health system relied on anticipatory budgeting and integration of climate change planning in the health budgets. During response and to support health systems recovery, the authorities diverted resources and equipment such as vehicles to support the response to extreme weather events. The other avenue for financing was non-governmental organizations and other partners including the private sector who supported some health system response activities and research. The districts are also required to allocate a small proportion of their revenue to climate mitigation efforts before approval of annual government funding. However, based on revenue collections, this is most times deprioritized during actual implementation.

*When planning*,* we have to consider some conditions that arise due to extreme weather conditions. So*,* we tend to budget something extra*. (Health services, Moroto district)

#### Enablers of Health Systems adaptation in the three districts

Several factors across the health system building blocks were identified as enablers of health system resilience as summarized in Table 3. At the leadership and governance level, the factors were the availability of policies, frameworks, and plans that prioritize health sector mitigation and adaptation, strong coordination and collaboration among sectors and stakeholders, and political will and prioritization among actors. For the health workforce, their availability, adequacy, technical capacity and effective supervision were highlighted. In terms of health information systems, having quality data and information on health and climate risks and vulnerabilities, robust early warning and surveillance systems and a high level of community engagement in mitigation and adaptation measures were highlighted.

*The other strength is that from the political wing*,* which provides political will. They will lobby for the funding needed. Recently for Kalibu Health Centre we decided to construct a maternity ward*,* but we had no land. The local councilor made efforts to get land. He fundraised and although we have not got it yet*,* we are in the process. Some political leaders have ambulances that support the health system.* (Administrator, Kasese district)

For essential medical products and technologies, the enablers were the availability and sufficiency of technology, the efficiency of the supply chain and the reliability of transport means to support the supply chain. On service delivery, having systems to ensure continuity of service delivery, a functional referral system, provision of a wide range of services, and strong community health systems were key enablers. On financing, the enablers were the availability and adequacy of resources and obtaining financial resources from other sources including non-governmental organizations, donors, and the private sector.

*There is a strong private sector and when we get challenges*,* there is a cement factory that sometimes gives us funds. We have banks as well as non-governmental organizations. Religious leaders also support us to pass information to the community. Then*,* we also leverage on the already existing structures in the district to reach the people* (Health services delivery, Kasese district)

**Table 3:** Enablers of health systems resilience

Theme	Sub-theme
Leadership and governance	• Clear policies, frameworks and plans that prioritize health sector mitigation and adaptation. • Strong coordination and collaboration among sectors and stakeholders. • Political will and prioritization among actors. • Clear administrative structures at facility and district levels. • Knowledge and awareness of leaders of their roles in building resilient health systems. • Ongoing committee planning, preparedness, and actions. • Support and involvement of community resources including leaders and community health workers. • Strong partnerships with private and non-governmental sectors. • Decentralization of response structures.
Health workforce	• Availability and adequacy of health workers. • Technical capacity of health workers to respond to and manage related health risks. • Effective supervision mechanisms. • Contingency plans for the deployment of sufficient health personnel for acute shocks.
Health information systems	• Data and information on related health risks, local vulnerabilities and impacts. • Robust surveillance systems to monitor related health impacts and trends. • Robust early warning systems for climate-related health risks. • High ease of propagation of information.
Essential medical products and technologies	• Availability and sufficiency of technology. • Efficiency of the supply chain. • Reliability of transport means to support the supply chain.
Service delivery	• Systems that ensure continuity of service delivery. • A functional referral system. • Provision of a wide range of health services. • Strong community health system. • Empowered community. • High level of community engagement in mitigation and adaptation strategies. • Good state of infrastructure and access to utilities.
Financing	• Availability and adequacy of resources. • Financial support from other entities including nongovernmental organizations, donors, and the private sector.

## Discussion

This study documented the impact of extreme weather events on health systems and their resilience in Bududa, Kasese, and Moroto districts in Uganda. Floods, landslides, and droughts, the major extreme weather events in the districts, impacted all building blocks of the health system including leadership and governance, human resources, infrastructure, drugs and supplies, and information systems affecting health services delivery to the community. The health systems employed several mechanisms to mitigate, adapt to, and recover from the climate shocks they experienced. The pre-response mechanisms ranged from development of policies, establishment of disaster committees, institution of early warning systems, training of health workers, and equipping district stores with drugs. During the response, the mechanisms included reassigning health workers to facilities, improving water and sanitation at facilities, relocating some facilities, engaging the community on potential diseases and risks, and mobilizing additional resources. To ensure recovery, the health systems conducted joint assessments to plan for future response, constructed and renovated health facilities, increased access to utilities, improved health worker supervision, and advocated for increased medical supplies. Overall, the enablers for building climate-resilient health systems were a clear policy framework including mechanisms for stakeholder coordination, political will, availability of resources, and availability and adequacy of health workers, and their technical capacity. An efficient supply chain, robust surveillance and strong early warning systems, high community engagement and a strong community health system were additional enablers. Whereas there are efforts to adapt by the health systems, the effects of extreme weather events usually overwhelm the system and response measures are usually slower in pace and many times are instituted after significant damage has been registered. Thus, effort is required to build preventive capacity and response to minimize the effects of extreme weather events and facilitate faster response and recovery. Several of the adaptation measures instituted also come along with unintended consequences that also affect the health system. Better preparedness and anticipation of these effects could help in strengthening the health system response and minimizing unintended consequences including negative impacts on communities.

The health sector can play a critical role in building resilience to climate change [18, 26]. However, like in most of sub-Saharan Africa, health systems in Uganda are plagued by poor leadership and governance, underfunding, inadequate human resources, fragmentation of roles, and overall poor health outcomes [27, 28]. The health systems are thus not prepared to deal with the increased burden due to climate change. In the study districts, extreme weather events damaged infrastructure and exerted pressure on health systems increasing the demand for health services while impacting their availability, accessibility, and quality in various ways [29], similar to previous reviews [30–32]. This calls for urgent mechanisms to build climate-resilient health systems, especially in regions that are disproportionately affected by climate change. To achieve this goal, areas that have frequently experienced extreme weather events such as Bududa, Kasese, and Moroto districts provide important lessons.

The WHO lists ten key components for building climate-resilient and low-carbon health systems linked to the six building blocks of the health system. In the districts, several efforts to ensure climate transformative leadership and governance were highlighted including establishing policies and frameworks that integrate health in climate change planning and of committees that routinely oversee disasters. Multisectoral disaster management committees with representatives from the health sector support mitigation, adaptation and recovery [5, 33–35]. Uniquely, in these districts, these committees were decentralized to the village level in vulnerable communities strengthening their role in supporting the health system. On building a climate-smart health workforce, district leaders highlighted training on climate and health to increase knowledge and awareness and strengthen their technical capacity for response. Recent studies have shown that significant gaps in knowledge and capacity exist among health workers [36, 37] and this area requires further efforts to bridge existing gaps. These efforts should start earlier with the integration of climate change and health into the curriculum [6, 36, 38, 39] which is lacking in low- and middle- income countries [39]. Other mechanisms should include the dissemination of information and data on climate change and health to health workers and engaging them in appreciating the implications, and collaboratively innovating response mechanisms. Other strategies were to ensure the availability and retention of health workers through incentives such as provision of accommodation facilities or transportation means to facilities. There were also efforts to post disaster-familiar staff to the prone areas to rely on their experience and increase retention. These measures go beyond those reported in previous reviews [5, 33–35] and require further contextualization and evaluation. The three sub-components of health information systems had some gaps that ought to be bridged. Generally, there is a need for more effort in assessing climate and health risks and greenhouse gas emissions. The Uganda Ministry of Health in 2024 concluded a vulnerability adaptation assessment and launched the health national adaptation plan [40], the second country to do so after Nepal. This plan will be instrumental in supporting health systems across the country to recognize major climate hazards and accordingly institute measures to minimize their impact on the population. Broader efforts are also needed to enhance early warning and surveillance systems and research in climate change and health at the national and sub-national levels. Systems should support tracking the impact of climate events on health and health systems and the identification of vulnerabilities for action. There is also a need to define a research agenda in climate change and health and support impactful research in this area that can inform policy and practice. Recently, efforts to integrate climate data and that from health information systems are ongoing in Uganda and will be instrumental in increasing access to information and conducting surveillance. Current district-level efforts in developing and reviewing early warning systems, forecasting climate and health risks, and surveillance of high-risk areas are commendable but emerging gaps should be bridged with support from the national level.

To build climate-resilient infrastructures, technologies, and supply chains, the districts implemented climate-resilient infrastructure and designs, equipped stores with medicines and increased the availability of emergency drugs. The other measures were to maintain road infrastructure to facilities and reliance on heavy-duty vehicles to ensure last-mile delivery. This is an area that has significant gaps, but with high potential to improve health system climate resilience and lower carbon footprint. It thus requires more attention. From previous studies, potential interventions have included the promotion of energy efficient infrastructure, improving housing insulation to reduce extreme indoor temperatures and ensuring thermal comfort through improved ventilation [38, 41]. Overall, as previously noted, infrastructural changes are more reported in high and middle- income countries compared to their low-income counterparts, whose interventions are more focused on community engagement [35]. To manage environmental determinants of health, most efforts were focused on water and sanitation at facilities and communities with less focus on other determinants such as air quality, housing, and food and nutrition. The gaps in health information systems impacted the integration of climate change in health programs. In terms of climate-related emergency preparedness and management, districts designated evacuation centers and facilities to handle specific conditions, upgraded facilities from health centers to hospitals, attached essential equipment including ambulances to facilities and increased outreaches to communities. For sustainable climate and health financing, districts reported several gaps, which they tried to bridge through anticipatory budgeting, integrating climate change planning in health budgets and mobilizing funds from non-governmental organizations and other partners. The districts also reported the diversion of other resources such as vehicles to support the health system during extreme events. One mechanism was to require districts to devote a certain proportion of their revenue to climate mitigation efforts before governmental funding is approved. However, during actual implementation, climate mitigation initiatives were not prioritized for funding and thus not implemented. This area requires rethought to have districts showing evidence of expenditure beyond budgetary allocation. The allocation of funds within the government-funded budget would also be significant as well as tapping into existing climate finance mechanisms [42].

The enablers of building climate-resilient health systems are similar to those previously reported [5, 33, 35]. Overall, health systems must enhance early warning systems, conduct climate risk assessments, ensure the availability of climate-resilient infrastructure and supply chains and enhance the technical capacity of health workers in climate change and health. There is also a need to utilize data sources to support climate change-informed programming and technology to enhance health services delivery and resilience, which were eminent gaps in the study districts. These enablers should guide initiatives to build climate-resilient health systems in similar contexts. Concrete examples of initiatives to build climate resilient health systems have been previously reported [5, 26, 30]. There is, however, the need for clear monitoring and evaluation systems to provide evidence of the effectiveness of the adaptation measures as well as research on their cost-effectiveness. The Lancet Countdown indicators to monitor national-level progress on climate change and health could serve as a good starting point [43] notwithstanding the current gaps that have impeded indicator monitoring [44]. In this study, health systems mostly employed measures to build resilience rather than low-carbon health systems in line with WHO guidance for low-performing health systems with low greenhouse gas emissions [25]. This area, however, will require further attention as the health system’s performance improves, though opportunities to get started exist [30, 35]. Uganda’s Health National Adaptation plan launched in 2024 aims to strengthen health systems resilience to climatic hazards. The operationalization of this plan should extend to the districts through multisectoral stakeholder committees that are charged with developing, implementing and evaluating potential strategies to build systems resilience and platforms created for experience sharing and learning.

This study considered a wide range of stakeholders from multiple sectors who shared their multidisciplinary expertise that informed this research. We also interviewed stakeholders from the national level, which increased richness and depth. The study also employed the WHO framework for building climate-resilient and low-carbon health systems [25] which aligned it with current developments. Nevertheless, to improve the framework application, it is important to provide clear assessment tools with clearly defined indicators and measurement scales that can be widely applied and can support cross-context comparisons. There is also a need to expound on the guidance for assessing low-carbon health systems with clear examples as the case is with the climate resilient ones. The framework should also ensure a clear balance between adaptation and mitigation measures, especially that most LMIC health systems prioritize adaptation compared with mitigation. As the climate events in these districts are seasonal, there was a heightened sense of awareness of previous and ongoing interventions, reducing potential recall bias. Related to this, the cross-sectional nature of the study limited reflection on the temporal shifts or the prolonged impacts of climate change on health systems. The transferability of the study findings may be limited to areas that experience extreme weather events, and the adaptation measures may not apply to some climate hazards. It is also not possible to tell the effectiveness of implemented measures which future research should address.

## Conclusions

The study established that landslides, floods, and droughts impacted all facets of the health system in the three districts through difficulty coordinating multiple players during response, causing injuries and deaths among health workers, damaging infrastructure, drugs, and information systems, and complicating service delivery. To adapt, the health system employed several strategies including instituting policies, early warning systems, capacity building for health workers, and stocking district stores with medicines and supplies. The other mechanisms were improving water and sanitation at facilities, relocating some facilities, engaging the community on potential diseases and risks and mobilizing funding support. Overall, factors such as a clear policy framework including mechanisms for stakeholder coordination, political will, availability of resources and health workers, high community engagement, and robust surveillance and strong early warning systems were key enablers for the health system in the three districts. These lessons should be integrated into strengthening the capacity of health systems to anticipate, respond to, cope with, and recover from extreme weather events.

## Supplementary Information

Below is the link to the electronic supplementary material.

Supplementary Material 1: Additional file 01: Key informant interview guide

Supplementary Material 2: Additional file 02: COREQ checklist

## Data Availability

The transcripts analyzed during the current study are available from the corresponding author on reasonable request.

## Abbreviations

LMICs: Low and Middle-Income Countries
KIs: Key informants
WHO: World Health Organization
