# Supplementary material for: Climate-resilient health systems in the face of extreme weather events in Uganda: a qualitative study in three high-risk districts
Source: BMC Health Serv Res. 2026 Aug 10;26:1540. doi: 10.1186/s12913-026-15326-z (PMC13741718; doi:10.1186/s12913-026-15326-z)
Supplement: Supplementary file 1 [file 12913_2026_15326_MOESM1_ESM.docx]

## **Key informant interview guide for district and health facility stakeholders**

1. What extreme weather events do you usually experience in the district?

- Where are these weather events localized?
- What are the potential explanations offered regarding the occurrence of these extreme weather events?
- Comment about the frequency of these extreme weather events currently compared to previous periods.

2. How do these extreme weather events impact the health of people in your district?

- What impact do these events have on the diseases experienced in the area/district? Which diseases tend to occur more often during these extreme weather events? Who are the most affected by these diseases? (*Note: explore separately per extreme weather event mentioned*)

3. How do these extreme weather events impact the health system in your district? By health system I mean how health services provision is organized and financed in your district.

- How do these events impact the health system considering the following factors: Leadership & governance, Financing, Essential medical products & supplies, Health workforce, Health Information systems, and Service delivery. (*Note: explore separately per extreme weather event mentioned*)

4. What mechanisms do health systems in your district employ to:
a) Mitigate (prevent) the adverse effects of extreme weather events
b) Adapt (cope) with the adverse effects of extreme weather events
c) Recover from the adverse effects of extreme weather event
d) What is done at the district and community levels and by whom?
e) What is done at the health facility level to increase the ability to reduce or minimize the adverse effects of extreme weather events? e.g., in terms of leadership and governance, financing, health workforce, infrastructure, early warning, assessing hazards, risks, and vulnerability, research etc.
f) What programmes or activities are being implemented to increase potential of the health system to prevent, cope and recover from the adverse effects of extreme weather events within the district / health facility? These could include activities by NGOs and the private sector. (what does the programme do and who funds it).
5. In your view, what are the barriers to building health systems mitigation (prevention), and adaptation (coping, and recovery mechanisms to deal with the adverse effects of extreme weather events in the district?

- What major gaps / weakness do you foresee in building such health systems within your district / region?
- What would you say are the key threats to building such health systems in your district or region?

6. In your view, what are the facilitators of building health systems mitigation, adaptation, and recovery mechanisms to deal with the adverse effects of extreme weather events?

- What are the major strengths in the district / region that can support building such health systems within your district / region?
- What are the key opportunities you foresee in building such health systems in your district or region?

7. To what extent are you as the health sector involved in climate change related activities in the district / region and vice versa. If the stakeholder is not from health-related sector ask: to what extent are you involved in activities geared towards building health systems with the capacity to adapt, mitigate, cope and recover from adverse weather events?
8. What recommendations do you suggest to support building health systems with the capacity to prevent, cope and recover from adverse weather events in your district / region / country?

- What should the ministry of health and other stakeholders do differently?
- What do you need to do differently at your level?

**For national level stakeholders**

1. What mitigation and adaptation mechanisms are employed at the national level to support the health system to deal with the adverse effects of extreme weather events? (Note: Only use probes after extensively exploring what is currently being done)

- **Leadership and governance** such as developing policies or programmes, integration of climate considerations in processes, policies, and plans or cross-sectoral collaboration to protect health from climate change.
- **Health workforce** such as training; information, awareness and communication; health workers taking steps to reduce their footprint.
- **Assessments of climate and health risks** to understand hazards, exposures, health risks and vulnerabilities, tracking progress.
- **Integrated risks monitoring, and early warning** such as Data on climate-sensitive environmental risks, hazards, and epidemiological trends, communicating information on hazards, exposures, health risks and vulnerabilities early, or timely warning communicated to health decision-makers, the media and communities.
- Conducting **research on climate and heath** e.g., establishing research agenda, building research capacity and translating research on climate and health into policies
- **Adaptation of current infrastructures, technologies, and supply chain** to current and future risks.
- Management of environmental determinants of health including monitoring of air and water quality standards, food security forecasting,
- **Climate-informed health programmes** such as equipping health facilities with Water and Sanitation for Health Facility Improvement Tool (WASH FIT), enhancing monitoring and water- and food-borne disease surveillance systems during high-risk periods (drought, flood, extreme heat) etc.
- **Climate-related emergency preparedness and management**, climate sensitive health risks to reduce vulnerability and exposure to climate hazards, Strengthen health system capacity to manage risks, and community empowerment to prevent and respond to risks.
- **Sustainable climate and health financing**, health specific funding and financing, access to financing by health sector

2. Considering Bududa, Kasese, and Moroto districts:

- What initiatives are implemented at the district and community levels and by whom?
- What is done at the facility level to increase climate resilience e.g., including leadership and governance, financing, health workforce, infrastructure, early warning, assessing hazards, risks, and vulnerability, research etc.
- What programmes are currently implemented to increase health system resilience in the country? (what does the programme do and who funds it).

3. In your view, what are the barriers to building health systems mitigation, adaptation, and resilience mechanisms to deal with the adverse effects of extreme weather events in the country?

- What major gaps / weakness do you foresee in building climate resilient health systems in Uganda?
- What would you say are the key threats to building climate resilient health systems in Uganda?

4. In your view, what are the facilitators of building health systems mitigation, adaptation, and resilience mechanisms to deal with the adverse effects of extreme weather events?

- What are the major strengths in the district / region that can support building climate resilient health systems in Uganda?
- What are the key opportunities you foresee in building climate resilient health systems in Uganda?

5. To what extent are you as the health sector involved in climate change related activities in the district / region and vice versa.
6. What recommendations do you suggest to support building climate resilient health systems in Uganda?

- What should the ministry of health and other stakeholders do?
